# A Two-Step A/D Conversion and Column Self-Calibration Technique for Low Noise CMOS Image Sensors

**DOI:** 10.3390/s140711825

**Published:** 2014-07-04

**Authors:** Jaeyoung Bae, Daeyun Kim, Seokheon Ham, Youngcheol Chae, Minkyu Song

**Affiliations:** 1 Department Semiconductor Science, Dongguk University, Seoul 100-715, Korea; E-Mail: sorcebaeru@dongguk.edu; 2 Samsung Electronics Co.Ltd, Kiheung 446-711, Korea; E-Mails: leisiz@dongguk.edu (D.K.); sh.ham@samsung.com (S.H.); 3 Department Electronic Engineering, Yonsei University, Seoul 120-749, Korea; E-Mail: ychae@yonsei.ac.kr

**Keywords:** CMOS Image Sensor (CIS), Two-Step Single-Slope ADC, column self-calibration, low noise

## Abstract

In this paper, a 120 frames per second (fps) low noise CMOS Image Sensor (CIS) based on a Two-Step Single Slope ADC (TS SS ADC) and column self-calibration technique is proposed. The TS SS ADC is suitable for high speed video systems because its conversion speed is much faster (by more than 10 times) than that of the Single Slope ADC (SS ADC). However, there exist some mismatching errors between the coarse block and the fine block due to the 2-step operation of the TS SS ADC. In general, this makes it difficult to implement the TS SS ADC beyond a 10-bit resolution. In order to improve such errors, a new 4-input comparator is discussed and a high resolution TS SS ADC is proposed. Further, a feedback circuit that enables column self-calibration to reduce the Fixed Pattern Noise (FPN) is also described. The proposed chip has been fabricated with 0.13 μm Samsung CIS technology and the chip satisfies the VGA resolution. The pixel is based on the 4-TR Active Pixel Sensor (APS). The high frame rate of 120 fps is achieved at the VGA resolution. The measured FPN is 0.38 LSB, and measured dynamic range is about 64.6 dB.

## 1. Introduction

Currently, the CMOS Image Sensor (CIS) is widely used in digital cameras, digital camcorders, CCTVs, medical equipment, *etc*. Among many kinds of CIS studies, R&D to improve the frame rates has been considered important. The frame rates are determined by the conversion speed of the Analog to Digital Converter (ADC) that exists in every column. These days, most CIS systems in a variety of applications use a Single-Slope ADC (SS ADC) because of a simple structure and excellent linearity [1]. However, such a system has a disadvantage in that the conversion speed of the SS ADC slows down at a rate of 2^n^ times in proportion to an increase in the resolution (n). Furthermore, this makes it difficult to adopt the SS ADC in high resolution systems including digital camcorders, HDTVs, UDTVs, or in other applications that require the image sensor to have frame rates faster than 30 fps. Many papers have reported attempts to improve the disadvantages of SS ADC [2–15]. Among them, cyclic ADC or Successive Approximation Register (SAR) ADC are well-known techniques for the high frame rate CIS. However, those methods require large chip areas and huge power consumption compared to SS ADC, thus it is difficult to apply them for portable devices such as mobile phones, digital cameras, *etc*. By the way, a Two-Step Single-Slope ADC (TS SS ADC) with a conversion speed faster by more than ten times faster than the SS ADC with the similar area and power consumption has been developed [2,3]. However, due to errors between the coarse ADC and the fine ADC, the fixed pattern noise (FPN) is a serious issue. Further, the error in the slope ratio between the coarse ramp and the fine ramp is one of the main causes of image quality degradation. Therefore, in order to eliminate the FPN, this study proposes a low noise CMOS image sensor with a Two-Step Single-Slope ADC and column self-calibration technique. The contents of this paper are as follows: In Section 2, the architecture and the circuit technique for Two-Step Single-Slope ADC are discussed. In Section 3, the circuit implementation for the proposed CIS is described. Measured results are described in Section 4. Finally, the conclusions are summarized in Section 5.

## 2. Two-Step Single-Slope ADC

### 2.1. Conventional Two-Step Single-Slope ADC (TS SS ADC)

**Figure 1.**
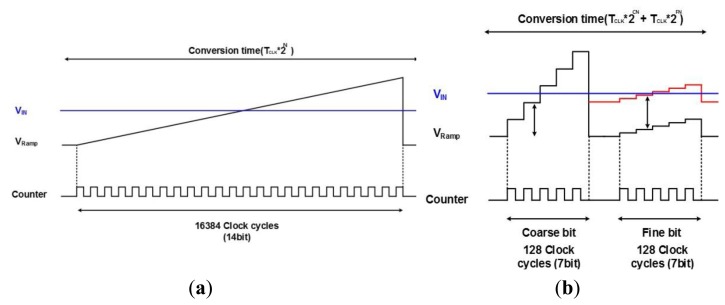
Principle of Single-Slope ADC and Two-Step Single-Slope ADC: (**a**) 14-bit single-slope ADC (SS ADC); (**b**) 14-bit two-step single-slope ADC (TS SS ADC).

Figure 1 shows the basic principle of SS ADC and TS SS ADC. The SS ADC shown in Figure 1a maximally requires 2^14^ (16,384) clock cycles in the worst case to satisfy the 14-bit resolution. Thus the operating speed is too slow to obtain a desired digital code in a high resolution ADC beyond 14-bit. On the contrary, the TS SS ADC is composed of both a coarse block which performs the conversion of the upper bit and a fine block which performs the conversion of the lower bit. For example, a 14-bit TS SS ADC shown in Figure 1b is separated by a coarse A/D conversion for the upper 7-bit and a fine A/D conversion for the lower 7-bit, respectively. Only 256 clock cycles is enough to obtain a 14-bit resolution, because there are 128 clock cycles at the coarse ADC and 128 clock cycles at the fine ADC. Hence, the operating speed of TS SS ADC is much faster (by 64 times) than that of SS ADC.

**Figure 2.**
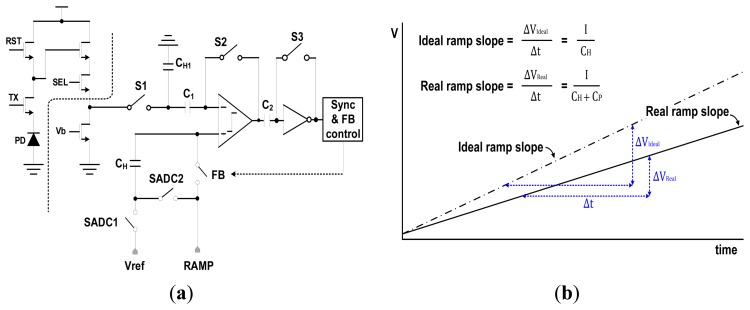
Circuit diagram and ramp slope change of TS SS ADC: (**a**) circuit diagram for a conventional TS SS ADC; (**b**) ramp slope change by parasitic capacitances.

Figure 2a shows the circuit diagram for an analog correlated double sampling (CDS) block with a conventional TS SS ADC [4]. It is composed of a comparator to compare the pixel signal with the ramp signal, four capacitors to perform the coarse A/D conversion and fine A/D conversion, a few switches, and a digital control block. From Figure 2a, the holding capacitor (C_H_) which stores the final coarse analog voltage is connected to the external ramp signals in a series. When the next fine ramp signal drives the comparator through the holding capacitor, the fine ramp slope is distorted by parasitic capacitances. Parasitic capacitances (C_P_) are inevitably formed by the switching MOS transistors, metal routing, and other side effects. Thus the real ramp slope is changed as follows:

(1) $$\text{Ideal}\;\text{Ramp}\;\text{Slope}=\frac{I}{C_{H}}\to \text{Real}\;\text{Ramp}\;\text{Slope}=\frac{I}{C_{H}+C_{P}}$$

Figure 2b shows the conceptual diagram of ramp slope change by the parasitic capacitances. It generates the difference of slope ratio between the coarse ramp and the fine ramp. Such difference in the slope ratio degrades the linearity of the ADC which is the most important part in the CMOS image sensor. Further, the column fixed pattern noise (CFPN) becomes serious because the parasitic capacitance is differently formed in every column. Normally, since the metal routing, device mismatching, and other side effects of each column are different, the value of parasitic capacitances of each column is also different. Thus the gain and linearity of each column ADC are also different. For those reasons, in case of the conventional TS SS ADC, it is very difficult to obtain a high resolution beyond 10-bit, and additionally, the linearity is much worse than that of SS ADC.

### 2.2. Proposed Two-Step Single-Slope ADC (TS SS ADC)

**Figure 3.**
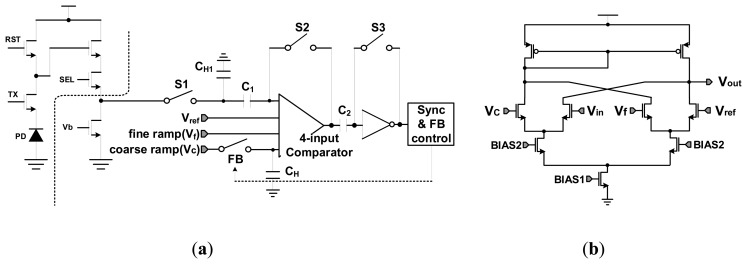
Circuit diagram for analog CDS with the proposed TS SS ADC: (**a**) analog CDS structure; (**b**) 4-input comparator.

Figure 3 shows the circuit diagram of the proposed TS SS ADC. From Figure 3a, the analog CDS block performs the A/D conversion with a new 4-input comparator, where the input nodes for the coarse ramp and the fine ramp are separated. It is different from that of the conventional TS SS ADC shown in Figure 2. Figure 3b shows the circuit diagram of the 4-input comparator based on the Gilbert cell structure. Normally, the ideal transfer function of Figure 3b is as follows:

(2) $$V_{\text{out}}=g_{m}\times R_{\text{out}}\times [(V_{\text{coarse}\_\text{ramp}}-V_{\text{in}})+(V_{\text{fine}\_\text{ramp}}-V_{\text{ref}})]$$

Where *g_m_* is the input transconductance, and *R_out_* is the output impedance. From Equation (2), the most important consideration of the Gilbert cell is the device mismatching of the input MOS transistors. Assuming that there is a device mismatching, the current flows are different at the input MOS transistors. In this case, the gain mismatching occurs between the coarse block and the fine block. Further, the Equation (2) has some errors. In order to solve the problems, a column self-calibration technique is proposed. It will be discussed later.

**Figure 4.**
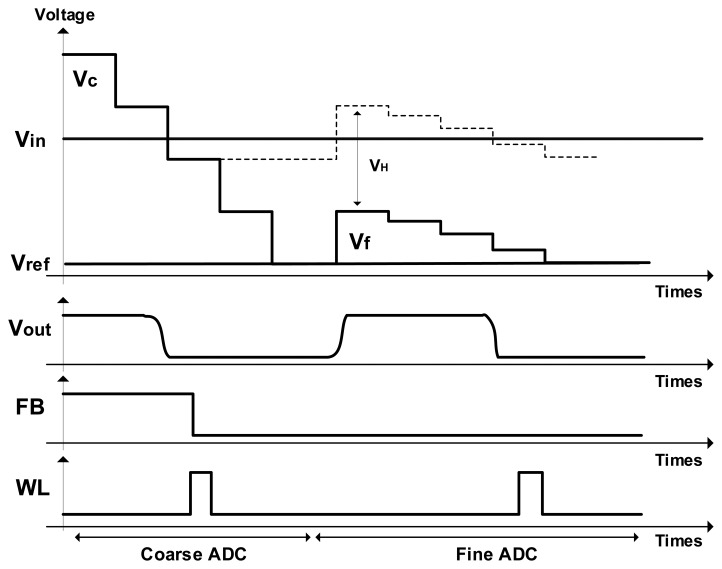
Timing diagram of the TS SS ADC **(**design example for 4-bit TS SS ADC: 2-bit coarse ADC and 2-bit fine ADC).

Figure 4 shows the timing diagram of the TS SS ADC. It is a simple design example for 4-bit TS SS ADC with the 2-bit coarse ADC and the 2-bit fine ADC. In order to easily understand the principle of TS SS ADC, we use a very simple 4-bit design example. Even though this procedure is almost equal to that of the conventional TS SS ADC, the proposed TS SS ADC has some advantages. First of all, the input stage of the proposed TS SS ADC is very simple because of the 4-input comparator. Since the switches of SADC1 and SADC2 at the conventional TS SS ADC are eliminated, the switching noises are reduced by about 30%. This is because the total number of switches at Figure 3 is four, while that of Figure 2 is six. Based on the smaller number of switches for the proposed TS SS ADC shown in Figure 3, some errors such as clock feedthrough, charge injection, and other temporal noises at the switching MOS's are reduced by about 30%, compared to the conventional TS SS ADC shown in Figure 2. Further, in order or reduce the errors, we use a large holding capacitance where the value of C_H_ is about 850 fF. From simulation results, the error at the large holding capacitance is very low to satisfy the 14-bit resolution, and the operating speed is enough to satisfy 120 fps. Secondly, very low offset errors generate because the voltage of Vref is directly connected to the input node of the comparator. Finally, the best advantage of the proposed TS SS ADC is an elimination of the serial holding capacitor shown in Figure 2. Since the holding capacitor is only used to hold the final voltage of the coarse ramp, the fine ramp slope is not affected by the parasitic capacitance. For this reason, the variation of fine ramp slope can be almost reduced.

## 3. Circuit Implementation

### 3.1. The Structure of CMOS Image Sensor

**Figure 5.**
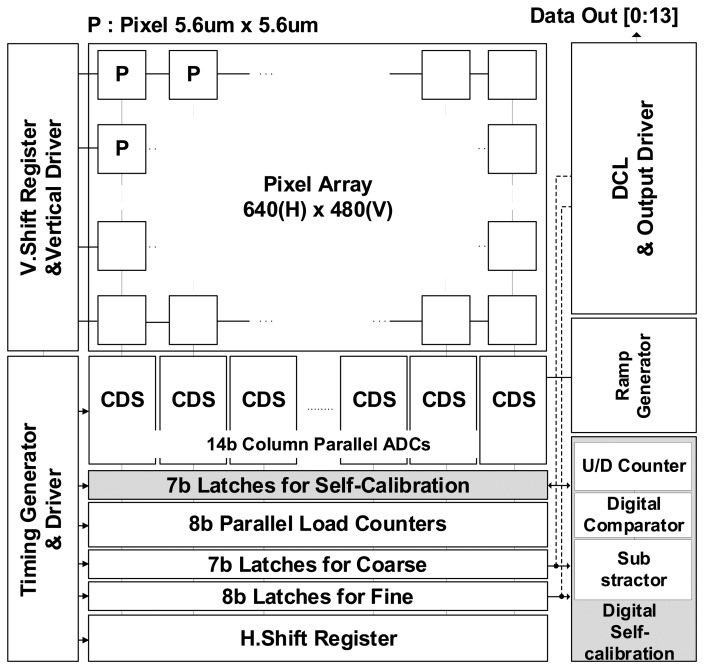
Structure of the CMOS image sensor with the 14-bit TS SS ADC.

Generally, a CIS with a column parallel ADC structure consists of a pixel array, a Correlated Double Sampling (CDS) block with a column parallel ADC, and a digital control block. The pixel array converts the light to the voltage, and the voltage is converted into a digital code through the CDS block. The digital control block plays a role in controlling the pixel, column, and output interface. Figure 5 shows the block diagram of the CIS with the 14-bit TS SS ADC. The CIS is based on the column-parallel structure with the VGA resolution of 640 × 480, and the pixel uses the 4-TR APS with a size of 5.6 μm × 5.6 μm. The column TS SS ADC consists of both one counter with the parallel load corresponding to the lower bit and two memories which store the digital values of the upper and lower bit. Additionally, it is designed with the memory for the self-calibration.

### 3.2. Design of a Digital CDS

**Figure 6.**
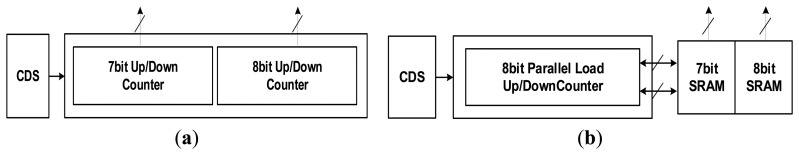
Block diagram of digital CDS: (**a**) conventional digital CDS; (**b**) proposed digital CDS.

The digital CDS performs a normal CDS processing by using a digital method, which means that the method of noise reduction is employed in a digital domain by converting the reset voltage and the signal voltage into a digital code. Figure 6a shows the block diagram for the conventional digital CDS. To implement a digital CDS, two up-down counters for the 7-bit coarse ADC and the 8-bit fine ADC are required as well as an analog CDS. The extra 1-bit of fine ADC is used for the digital correction logic (DCL) [8,14]. Such an up-down counter has an advantage in that it reduces unnecessary circuits, because the counter plays a role as the subtractor and memory itself. However, it is difficult to apply the up-down counter within a limited column pitch because the layout area of the counter is large. To compensate for this phenomenon, a digital CDS structure for the proposed 14-bit TS SS ADC is shown in Figure 6b. The structure is comprised of both an 8-bit up-down counter (including the DCL bit) with a parallel load which designates the initial value of the counter and two SRAMs. The SRAMs are designed with 7-bit and 8-bit. Since the layout area to implement the 1-bit is significantly smaller for the SRAM than for the counter, we can have a smaller size than that of the conventional structure by more than 10%.

**Figure 7.**
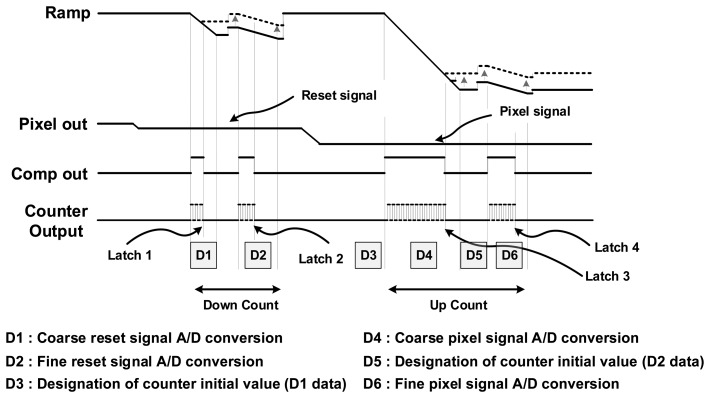
Timing diagram of the proposed digital CDS.

Figure 7 shows the timing diagram which illustrates the proposed digital CDS operation. First, the A/D conversion of the reset signal is performed at both the coarse block and the fine block, and the converted data values are stored to each SRAM. After that, the digital values stored in the memory are set to the initial values of the counter. Then, from the initial values, the counting starts in reverse order of the reset signal, in order to perform the same action with the conventional up-down counter. Finally, the A/D conversion of the signal voltage is performed at both the coarse block and the fine block.

### 3.3. Column Self-Calibration Technique

**Figure 8.**
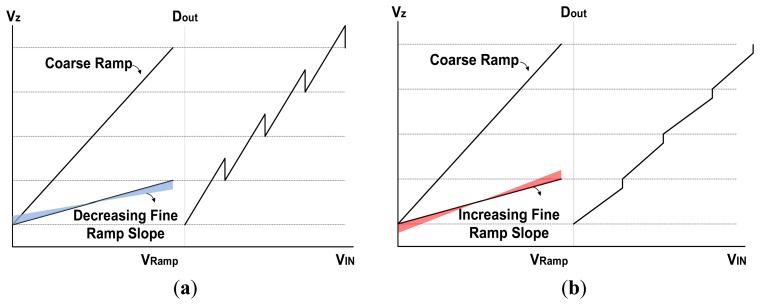
The error of output digital code depending on the slope change of fine ramp: (**a**) decrease of fine ramp slope; (**b**) increase of fine ramp slope.

As was previously mentioned, the difference in the slope ratio between the coarse ramp and the fine ramp generates large amounts of CFPN that degrades the image quality. Figure 8 shows the errors of the digital output data that depend on the slope ratio of the two ramps. To compensate such a problem, this paper proposes a new 4-input comparator. However, the error of ramp slope in the comparator may not be perfectly compensated by the 4-input comparator. Generally, the ramp slope ratio of TS SS ADC is determined by the resolution of coarse ADC and fine ADC. It is given by:

(3) $$S_{C}:S_{F}=\frac{T_{C}}{2^{N_{C}}}:\frac{T_{F}}{2^{N_{C}+N_{F}}}$$

where S_C_ and S_F_ are the ramp slope of coarse ADC and fine ADC, T_C_ and T_F_ are the conversion time of coarse ADC and fine ADC, N_C_ and N_F_ are the resolution of coarse ADC and fine ADC, respectively. If the ramp slope ratio in Equation (3) differs from the ideal value, as shown in Figure 8, the TS SS-ADC has a non-linearity performance because of the over codes or missing codes. Therefore, a self-calibration circuit to correct the ramp slope error in the comparator must be designed.

**Figure 9.**
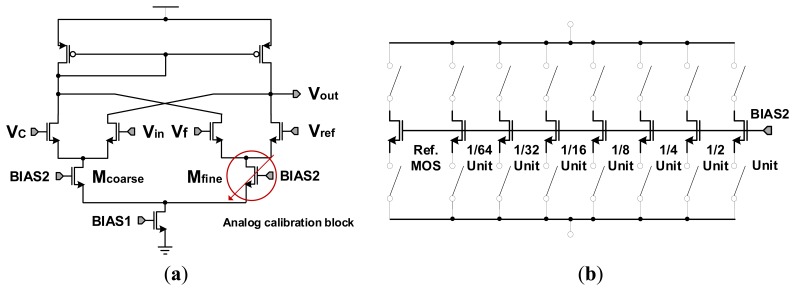
Circuit diagram of the self-calibration technique: (**a**) circuit diagram of 4-input comparator; (**b**) magnified circuit diagram for M_fine_.

Figure 9a shows the circuit diagram of the 4-input comparator with a self-calibration. Here, the currents at M_coarse_ and M_fine_ are not the same due to the mismatching of the two MOSs, causing an uneven analog gain and a difference in the slope ratio between the coarse ramp and the fine ramp. The study is focused on the analog calibration of M_fine_ that we change the gm value by adjusting the current amount on M_fine_, in order to make the same analog gain at M_coarse_ and M_fine_.

Figure 9b shows the magnified circuit diagram for M_fine_. The gm value at M_fine_ can be adjusted by trim SRAM signal in the digital calibration block. The size of trimming transistors M_fine_ and the bit resolution of feedback circuit are the most important factors to decide the effective range and accuracy of the self-calibration. In order to solve the problems, we have analyzed the error range of each column ADC with Monte-Carlo simulation. From the analysis results including device mismatching errors, we found that the maximum error code was 63LSB, namely 6-bit resolution. Thus a self-calibration circuit with a 7-bit resolution and a control circuit with 0.5LSB step are designed to compensate the 6-bit code errors.

**Figure 10.**
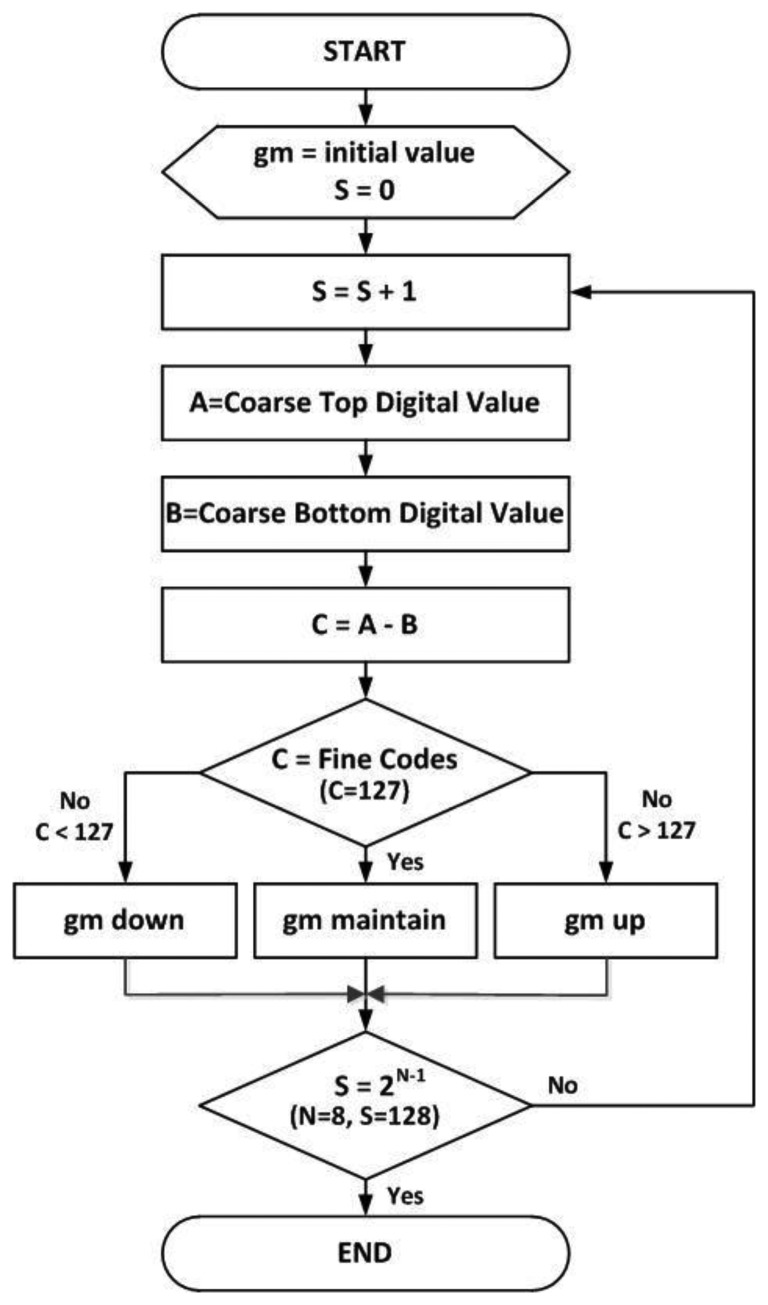
Flow chart of proposed self-calibration technique.

Figure 10 shows the flow chart of the proposed self-calibration technique. The slope correction between the coarse ramp and the fine ramp is performed based on the flow chart. In the proposed TS SS ADC, the total codes of fine ADC are 256, because we use a DCL technique with the extra 1-bit of coarse ADC shown in Section 3.2. Thus the fine ramp covers the 2-bit LSB of coarse ADC. It means that the maximum range of fine ramp is 256, which is larger than 1LSB of coarse ADC. Therefore, it is possible that C = A − B can be smaller or larger than 127, as shown in Figure 10.

**Figure 11.**
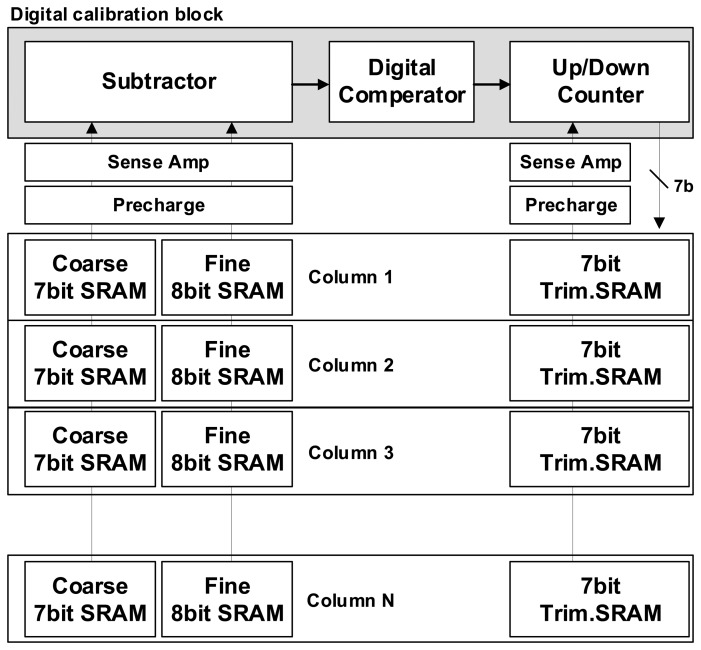
Block diagram for the self-calibration at the digital block.

Figure 11 shows the block diagram for digital calibration. First, the initial values configured as default in the trim SRAM are entered into the analog calibration block to start the self-calibration. Then, the analog CDS switch is controlled to convert the lowest fine value of the coarse 1 LSB into the digital code and it is stored to the first memory. Then, the highest fine value of the coarse 1 LSB undergoes the A/D conversion and is saved to the second memory. The two data are subtracted by the subtractor. Here, the difference between the two data causes a change of the initial value configured in the trim SRAM, depending on whether the value is smaller or larger than the ideal 7-bit fine code value (127). The digital code transition in the trim SRAM changes the total amount of the current flowing in the analog calibration circuit and changes the gm value of M_fine_. Repeating such methods finally makes the analog gain between M_coarse_ and M_fine_ the same. As a result, we reduce drastically the CFPN caused by the difference in the analog gain between the coarse ramp and the fine ramp with the device mismatching error. The calibration technique used in this work is one of a foreground method. The foreground calibration is that all the column calibration has been done before the beginning of CIS operations. Thus there is no lowering of frame rate because of the foreground calibration. However, it takes a time for the calibration. Based on the measured results in this paper, it takes 1.25 ms for VGA pixels. Of course, the more the number of pixels is increased, the more the foreground calibration time is also increased. Nevertheless, most of the recent foreground calibrations are sufficiently finished during the CIS ready time, even in a huge pixel system.

**Figure 12.**
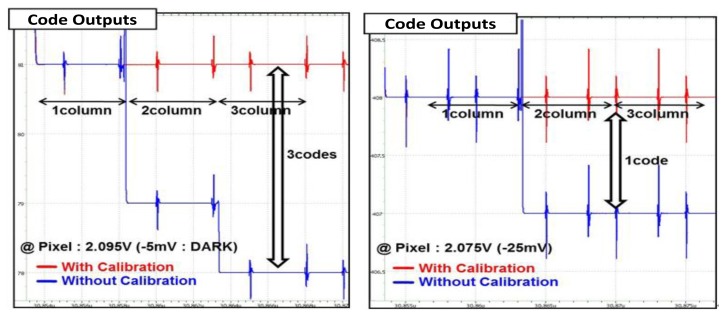
Simulation results for the self-calibration technique.

Figure 12 shows the simulated output values of the CIS with or without the self-calibration technique. We assume that the same input voltages are applied for each of the three columns with arbitrary mismatching error. For example, we assume that the pixel voltage is 2.095 V at the left figure, and it is 2.075 V at the right figure. From the simulation results, the code outputs of the image sensor without the self-calibration show different outputs for the three columns, even though the same input voltages are applied. It is said that the CFPN generates if the outputs for each column are different. However, we found that the three columns have the same output values, when the self-calibration technique is used. Therefore, the column self-calibration technique drastically reduces the CFPN.

## 4. Experimental Result

### 4.1. Chip Microphotograph and Measurement Systems

**Figure 13.**
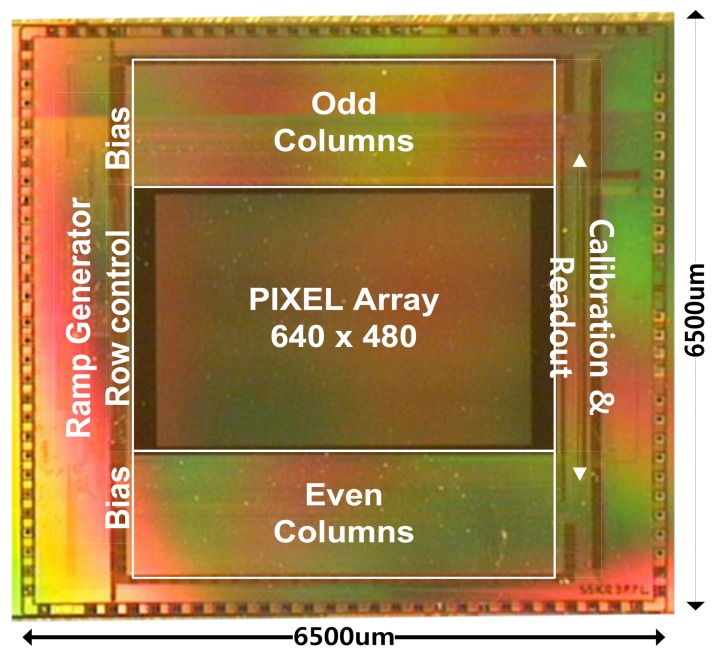
Microphotograph of the fabricated CIS with Samsung 0.13 μm CIS technology.

Figure 13 shows the chip microphotograph of the fabricated CIS with Samsung 0.13 μm 1P4M CIS technology. The chip size is 6.5 mm × 6.5 mm and the pixel array conforms to the VGA resolution (640 × 480). The CIS in this paper is configured to provide most of the control signals through an external FPGA. Using such a configuration allows us to establish various test environments for the image sensor, to verify the performance of the CIS, and to check its various features.

**Figure 14.**
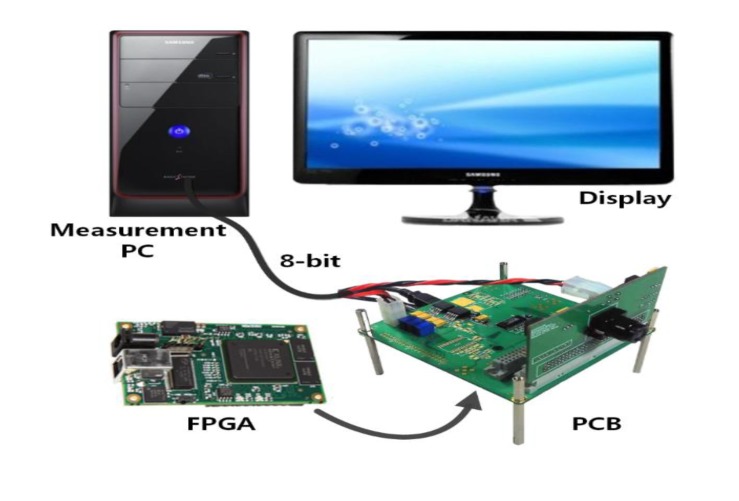
Configuration of measurement systems.

Figure 14 shows the configuration of measurement systems, and it is comprised of the board which contains the Xilinx-XEM 3050 FPGA and the board with the CIS of the Chip On Board (COB). The FPGA plays a role in generating the control signal for measurement, receiving the output data from the image sensor, and delivering the result to the PC through the USB interface. The transmitted data are handled in the PC to apply the processing of a real image. Here, unfortunately, the resolution of normal display is limited to 8-bit. Since the normal display systems such as LCD, LED display, and other ones are set to always 8-bit resolution, the testing for the 14-bit CIS has a problem. With a normal display, the 14-bit full-resolution of CIS cannot be tested. In order to overcome the problems, we divide and select the digital output codes of TS SS ADC from the FPGA one by one. For example, if we choose 7-bit coarse and 1-bit fine, the testing resolution is 8-bit. If we choose 3-bit coarse and 5-bit fine, the testing resolution is 12-bit. Assuming x-bit coarse ADC and y-bit fine ADC, we can obtain the testing resolution as follows:

(4) $$\text{Testing}\;\text{Resolution}=2^{7-x}\times 2^{x}\times 2^{y}=2^{7+y}$$

Therefore, (7 + y)-bit is the final testing resolution. From Equation (4), for example, the testing resolution is 14-bit, when we choose 1-bit coarse and 7-bit fine.

### 4.2. Measured Results and Images

**Figure 15.**
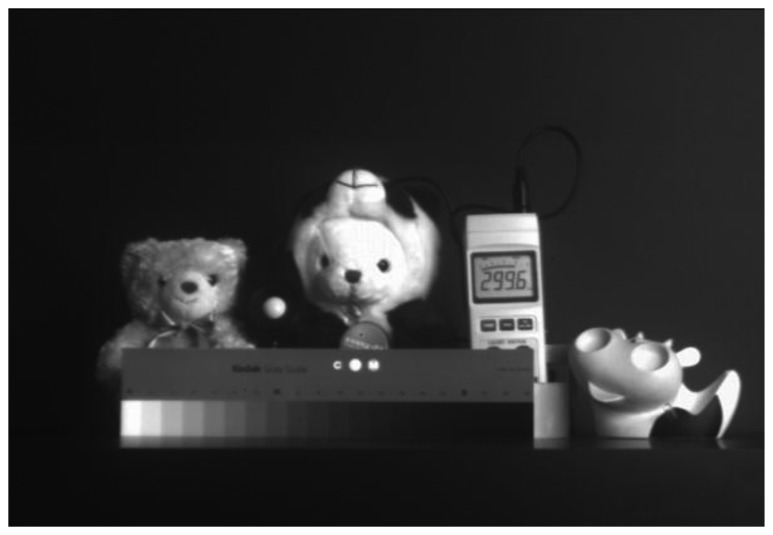
Measured VGA sample image with the 7-bit coarse and 1-bit fine (8-bit resolution).

**Figure 16.**
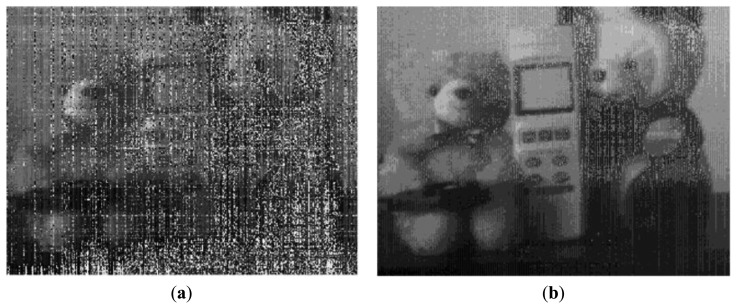
Measured sample image with the 1-bit coarse and 7-bit fine (14-bit resolution): (**a**) without the self-calibration; (**b**) with the self-calibration.

Figure 15 shows the measured VGA sample image with the condition of 7-bit coarse and 1-bit fine, namely 8-bit resolution. It achieves the frame rates of 120 fps at a main clock speed of 40 MHz. It shows a high image quality. Figure 16 shows the measured sample images with the condition of 1-bit coarse and 7-bit fine, namely 14-bit resolution. Figure 16a shows the sample image without the self-calibration technique. It shows very poor image quality due to the presence of high levels of random noise and of the CFPN caused by the degraded ADC linearity error from the analog gain difference between the two ramps. On the contrary, Figure 16b shows the sample images with the self-calibration technique. The ADC linearity is drastically improved as a result of the self-calibration of the analog gain difference between the two ramps. Therefore, the CFPN and the random noise in the image sensor were significantly reduced and an improved image quality was achieved.

**Figure 17.**
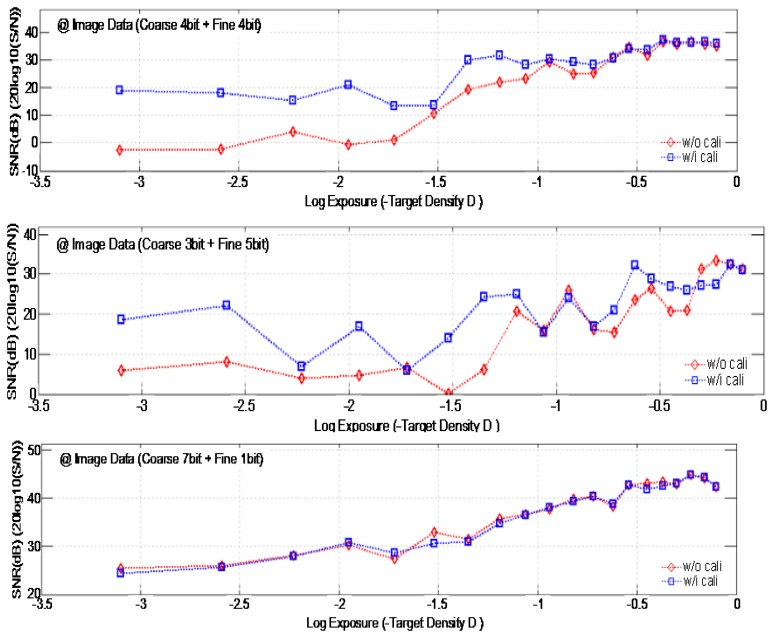
Measured SNR with/without the self-calibration.

Figure 17 shows the measured SNR dependent on the amount of exposure with/without the self-calibration. The measurement uses the TE241-OECF noise test chart and the analysis is performed by using the IMATEST software program. If the image data is achieved with the condition of 7-bit coarse and 1-bit fine (namely, 8-bit ADC), the fine bit data is included into the only 1-bit. Thus there is not a large difference in the SNR before and after the self-calibration despite the analog gain difference between the two ramps. If the fine bit data increases to a 4-bit or 5-bit level, the difference of the analog gain between the two ramps degrades the ADC linearity more, and the SNR without the self-calibration becomes very low. If we use the self-calibration technique, however, it improves the SNR about by more than 10dB. Specially, the effect is more excellent at the low exposure region.

**Figure 18.**
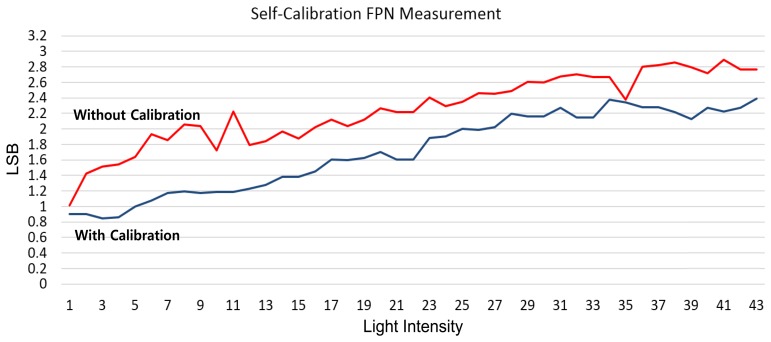
Measured CFPN with/without the self-calibration (10-bit resolution).

**Figure 19.**
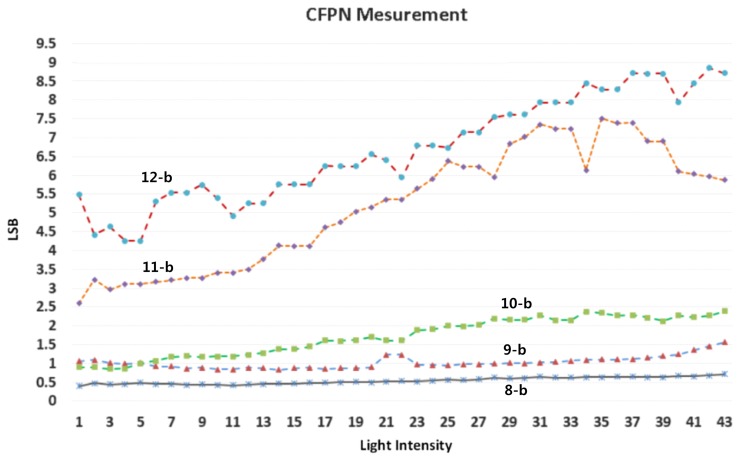
Measured CFPN with the self-calibration for various resolutions (scale of *y*-axis: 10-bit resolution).

**Figure 20.**
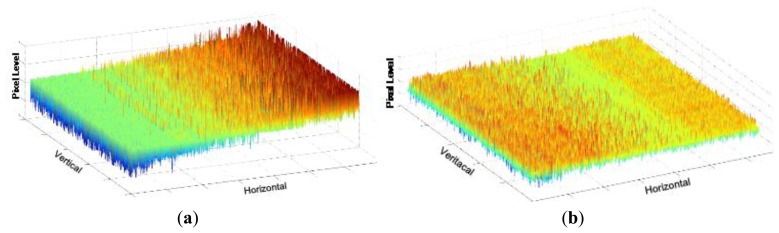
Measured results for the variation of pixel level: (**a**) without the digital CDS; (**b**) with the digital CDS.

Figure 18 shows the measured CFPN dependent on the light intensity before and after the self-calibration at the 10-bit resolution. The average CFPN is reduced by about 0.4 LSB after the self-calibration. Figure 19 shows the measured results of the CFPN with the condition of the n-bit coarse and m-bit fine. Since the normal display system like a LED display is set to the 8-bit resolution, the addition of n-bit and m-bit is always 8. For example, the measured condition of the 7-bit coarse and 1-bit fine is 8-bit resolution, and the measured condition of the 3-bit coarse and 5-bit fine is 12-bit resolution. The measured CFPN of 8-bit ADC is 0.38 LSB, and the measured CFPN of 12-bit ADC is 5.5 LSB, respectively, at the dark region for the 10-bit resolution scale. Even in 13-bit or 14-bit, the measured results of CFPN cannot be obtained. Therefore, the measured performance of this work is about 10.5-bit, even though a 14-bit CIS was designed. The reasons why the measured result of 10.5-bit is obtained will be discussed in the conclusions. Figure 20 shows the measured results for the variation of the pixel level with/without the digital CDS shown in Figure 6. The pixel output level without the digital CDS becomes uneven and results in a high CFPN. However, the overall pixel output level becomes even when the digital CDS is used, and the CFPN is decreased.

## 5. Conclusion

This paper described a CMOS image sensor with a Two-Step Single-Slope ADC, a high speed frame rate of 120 fps, a low fixed pattern noise (FPN), and a column self-calibration technique. In order to satisfy the required specifications, therefore, new techniques have been proposed in this paper. The TS SS ADC performed the A/D conversion by separating the coarse ADC and fine ADC, and it was suitable to implement the high speed CIS. However, the difference of ramp slope ratio between the coarse ADC and the fine ADC due to the device mismatching error degraded the ADC linearity and generates high levels of noise. To overcome such problems, this paper used a new 4-input comparator to prevent the changes in the ramp slope ratio and to remove the serial capacitor. Further, a feedback circuit of the self-calibration was designed to correct the difference of slope ratio between the coarse ramp and the fine ramp for each column, to improve ADC linearity, and to reduce CFPN generation. As a result, the SNR was improved by about 10 dB and the CFPN was reduced by more than 0.4 LSB relative to those of the TS SS ADC without self-calibration. The chip has been fabricated with the Samsung 0.13 μm 1P4M CIS technology. The resolution of the CMOS image sensor conformed to the VGA specifications of 640 × 480, and the pixel size was 5.6 μm with the 4-TR APS. The conversion time of the designed TS SS ADC satisfied 12.5 μs at the main clock speed of 40 MHz. Thus the frame rate was faster by about 48 times than that of the conventional one based on SS ADC. Table 1 shows the summary of measured CIS performance. Even though a 14-bit TS SS ADC was designed, the measured Effective Number of Bits (ENOB) in this CIS was about 10.5-bit from Figure 19 and Table 1. There were a few reasons why a low ENOB was obtained. First of all, a high performance ramp generator beyond 14-bit was not supported. Although a 14-bit ramp generator with a current steering DAC was designed, the measured ENOB was just 12-bit. In order to obtain a 14-bit TS SS ADC, the ENOB of ramp generator should have been the level of 16-bit. Secondly, kT/C noise, charge injection noise, and clock feethrough noise from switching MOS transistors were not carefully analyzed. They were the dominant noises to degrade the performance of ADC. Further, the pixel noise was also a problem to degrade the performance of CIS.

Table 2 shows the comparison results of the proposed ADC with the published works. In terms of FOM, while it is larger than that of SAR ADC [8], Delta-Sigma ADC [9], cyclic ADC [10], and Single-slope ADC [11], it is smaller than that of SAR/SS ADC [13], TS SS ADC [14], and TS SS ADC [15]. Even though the measured ENOB is 10.5-bit, the FOM performance of this work is remarkable in the area of TS SS ADC. Based on those conclusions, further works will be discussed. Firstly, a novel calibration technique based on a background method will be studied. In this work a foreground calibration technique has been proposed and verified, but a more efficient background calibration technique will be needed for the market products. Secondly, a high performance ramp generator beyond the 16-bit resolution will be proposed for the 14-bit TS SS ADC. As mentioned above, the performance of a ramp generator is the most critical point to obtain the desired results of 14-bit CIS. Thus the design of a 16-bit ramp generator will be focused on. Thirdly, the noise analysis must be researched in the area of pixels, analog circuits, and digital circuits, respectively. Further we have to analyze kT/C noise, charge injection noise, clock feethrough noise, and so on. Based on the results of noise analysis, in order to implement a 14-bit CIS, a few kinds of noise reduction techniques will be discussed.

## Figures and Tables

**Table 1.** Summary of the measured CIS performance.

Process technology	0.13 μm 1P4M CIS process
Chip size	6.5 mm × 6.5 mm
Core size	6 mm × 6 mm
Number of effective pixel	640 × 480 pixels
Pixel type	Non-shared 4T (pinned-photodiode)
Operating voltage	2.8 V(pixel)/2.8 V(analog)/1.5 V (digital)
Frame rate	120 fps (@40MHz)
ADC resolution	14-bit (measured ENOB: 10.5-bit)
P-FPN/C-FPN	0.43 LSB/0.38 LSB (@dark)
Dynamic range	64.6 dB
Power consumption	98 μW/column
Full well capacity	23,000*e*^−^
Conversion gain	43uV/*e*^−^
Total RN	$13.5e_{\text{rms}}^{-}$
Figure of Merit	43.9*e*^−^nJ

**Table 2.** ADC performance comparison.

**Reference**	**[8]**	**[9]**	**[10]**	**[11]**	**[13]**	**[14]**	**[15]**	**This work**
Technology	0.13 μm CIS	0.13 μm CIS	0.18 μm CIS	90 nm CIS	0.18 μm CIS	0.35 μm CIS	0.25 μm CIS	0.13 μm CIS

ADC [Type]	ΔΣ	SAR	Cyclic	Single-slope	SS / SAR	TS SS	TS SS	TS SS

A/D Digitizing Phases	N/A	N/A	N/A	N/A	3b-SS 8b-SAR	5b-coarse 6b-fine	6b-coarse 5b-fine	7b-coarse 8b-fine

Reported Resolution [bit]	>12	14	13	14	11	10	11	10.5 (measured ENOB)

Conversion Time [us]	2.3	1.7	2.3	7.4	12	4	2	3.2

1-H Time [us]	6.85	9.2	6	7.716	35	5.95	4	12.5

Power [uW]	55	41	300	300	32	150	170	68

ADC Figure of Merit (FOM)*[fJ]	15	4.2	84	135	187	292	166	150

* $\text{ADC}\;\text{FoM}=\frac{\left[\text{Power}]\times [\text{Conversion Time}\right]}{2^{\text{ENOB}}}$.
